# Pediatric Videosomnography: Can Signal/Video Processing Distinguish Sleep and Wake States?

**DOI:** 10.3389/fped.2018.00158

**Published:** 2018-06-19

**Authors:** A. J. Schwichtenberg, Jeehyun Choe, Ashleigh Kellerman, Emily A. Abel, Edward J. Delp

**Affiliations:** ^1^Department of Human Development and Family Studies, College of Health and Human Sciences, Purdue University, West Lafayette, IN, United States; ^2^School of Electrical and Computer Engineering, Purdue University, West Lafayette, IN, United States

**Keywords:** signal processing, pediatric, infant, sleep, toddler, actigraph, waking

## Abstract

The term videosomnography captures a range of video-based methods used to record and subsequently score sleep behaviors (most commonly sleep vs. wake states). Until recently, the time consuming nature of behavioral videosomnography coding has limited its clinical and research applications. However, with recent technological advancements, the use of auto-videosomnography techniques may be a practical and valuable extension of behavioral videosomnography coding. To test an auto-videosomnography system within a pediatric sample, we processed 30 videos of infant/toddler sleep using a series of signal/video-processing techniques. The resulting auto-videosomnography system provided minute-by-minute sleep vs. wake estimates, which were then compared to behaviorally coded videosomnography and actigraphy. Minute-by-minute estimates demonstrated moderate agreement across compared methods (auto-videosomnography with behavioral videosomnography, Cohen's kappa = 0.46; with actigraphy = 0.41). Additionally, auto-videosomnography agreements exhibited high sensitivity for sleep but only about half of the wake minutes were correctly identified. For sleep timing (sleep onset and morning rise time), behavioral videosomnography and auto-videosomnography demonstrated strong agreement. However, nighttime waking agreements were poor across both behavioral videosomnography and actigraphy comparisons. Overall, this study provides preliminary support for the use of an auto-videosomnography system to index sleep onset and morning rise time only, which may have potential telemedicine implications. With replication, auto-videosomnography may be useful for researchers and clinicians as a minimally invasive sleep timing assessment method.

## Introduction

The term videosomnography (VSG) captures a range of video-based methods used to record and subsequently score sleep behaviors. One of the first documented uses was at Brown University as Dr. Thomas Anders and colleagues attempted to record sleep in infants born preterm without the use of electrodes (1). Shortly after this, video recordings became a more common component of polysomnography (PSG). Over time, PSG with video became a field standard, and the additional informaton provided by these videos proved to be a valuable interpretation tool for sleep disorders across several populations [e.g., (2)]. However, until recently, the use of VSG (outside of PSG) has been primarily limited to research. With recent technological advancements and the rise of telemedicine approaches, the use of home-based VSG is increasing. Minimally invasive sleep methods, like VSG, are quickly being adopted by mass market devices, although few studies have tested the accuracy and feasibility of an automated-VSG approach.

Thus, to develop and subsequently test an automated VSG scoring system, this study builds on two lines of research: VSG and video signal processing. In the following sections, we will first review VSG (outside of PSG) followed by advances in signal processing that have led to the possibility of auto-VSG.

### Videosomnography research and clinical applications

Sadeh (3) described VSG as a sleep assessment best practice, noting its ability to non-invasively capture sleep, while also documenting some parasomnias and caregiver actions. Behavioral VSG includes human coding of sleep behaviors (e.g., wakings) by watching video recordings of sleep. However, behavioral VSG coding is time consuming and cannot accurately detect sleep if a child moves out of the video frame. Additionally, there are privacy concerns as most videos cannot be deidentified. These factors have limited the application of VSG in clinical settings, and thus VSG has primarily served as a research tool. Within a research setting, students are typically trained to code target behaviors (e.g., night wakings) over the course of 2–3 months. Once trained, behavioral coding in 4:1 time takes approximately 1 h per night of sleep. Despite this time consuming nature, previous studies have used behavioral VSG coding to generate estimates of quiet sleep, active sleep, wake after sleep onset (WASO), sleep position, caregiver behaviors, bed sharing, and infant crying or self-soothing behaviors (1, 4–9). The use of VSG has grown exponentially in the past 5 years and the larger sleep field would benefit from a standardized and more efficient VSG coding method. Fortunately recent advancements in video/signal processing may make this possible.

### Video signal processing

Within the field of electrical and computer engineering, the application of signal processing techniques to digital video data are common; however, the application to sleep is relatively novel. One of the first studies used infrared-based cameras to index motion during sleep (10). Subsequent studies used frame-by-frame differencing to extract activity, with the assumption that the sleeping individual was the only source of ‘difference’ or activity (11–13). Within these studies, VSG data from five children and ten adults were used to calibrate the sleep data extracted from the videos. A similar differencing method was then applied in a study of six children with Attention Deficit/Hyperactivity Disorder (14). Additional research used variations of this image differencing approach, while focusing on large/gross motor movements (15), head and trunk identification (16, 17), and infant sleep (18). Spatio-temporal prediction has been applied to VSG data to extract estimates of sleep and wake time (19). Preliminarily, deep learning has also been applied to VSG data in infants (20); although to date, no peer-reviewed articles have been published. Commercially available products like Nanit®, AngelCare®, BabbyCam® and Knit Health have quickly adopted automated VSG procedures, but minimal validation or clinical guidelines exist.

Within the present study, we build on the most established signal processing approach—specifically a frame-by-frame differencing approach, and apply it to existing pediatric VSG data. We aim to demonstrate how a signal/video-processing system can be used to generate estimates for sleep vs. wake states, in addition to estimates of sleep timing (sleep onset and morning rise time) and nighttime sleep duration. This study will provide valuable information about the assumptions, accuracy, and feasibility of automated-VSG scoring, to better inform our understanding of its use in both mass market devices and future clinical applications.

## Methods

### Participants

As a part of a larger longitudinal study on sleep in early development, families recorded their infant/toddler's sleep between 8 and 30 months of age (*M* = 17.8, *SD* = 6.1; Table 1). To enroll in the larger study infants/toddlers had to be a younger sibling of a typically developing child and the family's primary language was English. Exclusion criteria include severe visual, hearing, or motor impairment, or a fragile health condition, and the use of medications known to affect sleep or attention at time of enrollment (Table 1).

**Table 1:** Sample demographic characteristics (*N* = 30).

***Characteristics***	**Range, *M*(*SD*) or *n* (%)**
**INFANT/TODDLER**	
Sex (% Male)	19 (63%)
Age (months)	8–30, *17.8 (6.1)*
**Race and Ethnicity**	
White, Non-hispanic or Latino	25 (83%)
Black, Non-hispanic or Latino	1 (3%)
Multi-Racial	1 (3%)
Hispanic or Latino	3 (10%)
**MATERNAL AND HOUSEHOLD**	
Maternal age (years)	20–42, *31.5 (4.9)*
**Maternal Education**	
High School/GED	2 (7%)
College degree	16 (53%)
Graduate degree	4 (13%)
Other	6 (20%)
Unreported	2 (7%)
**Paternal Education**	
Some High School	1 (3%)
High School/GED	1 (3%)
College degree	12 (40%)
Graduate degree	8 (27%)
Other	6 (20%)
Unreported	2 (7%)
Marital status (% married)	27 (93%)
**Family Income**	
Below $20,000	1 (3%)
$20,001–$40,000	4 (13%)
$40,001–60,000	5 (17%)
$60,001–$80,000	6 (20%)
$80,001–$100,000	7 (23%)
$100,001 and above	6 (20%)
Unreported	1 (3%)

*Parents completed the family demographic form at the time of enrollment. Three families did not provide information on family income and one family did not provide paternal education information*.

For analyses one night per infant/toddler was selected in a quasi-random fashion (within this study quasi-random means that data selection was based on two practical factors, described below). The first 30 families that completed behavioral VSG and actigraphy coding were included. Only 30 families (of the overall 90 enrolled in the larger study) were included for two reasons. First, this longitudinal study and its coding are ongoing and the included families reflect those with behaviorally coded data at the time of analyses. Second, including 30 infants/toddlers with more than 500 min of “sleep vs. wake” data points per infant/toddler had sufficient power to detect moderate effect sizes (*d* = 0.50) with two-method differences and kappa agreement (power <0.95).

### Measures

#### Child demographic information

Infant sex, race, ethnicity, maternal education, paternal education, and family income were reported at time of enrollment.

#### Videosomnography

Video recordings of sleep were captured using a portable, night-vision camera (Swann SW344-DWD, Model ADW-400) that was placed over the infant/toddler's primary sleep location. Videos were processed using both behavioral codes and automated VSG coding methods, as outlined below.

#### Behavioral coding

First, each video was coded for sleep onset, morning rise time (sleep offset), and nighttime wakings. Wakings had to last longer than 1 min and include purposeful actions from the infant/toddler (e.g., sitting up, looking around, crying). Wakings were assessed in three ways: WASO, minor wakings, and major wakings (definitions provided in Table 2). Research assistants received over 40 h of training, including guided coding, practice videos, and monthly meetings with a trained coding lead. All assistants also completed up to two reliability training sets including five videos each. For the current sample, inter-rater reliability exceeded our predetermined intraclass correlation coefficient (ICC > 0.70) threshold for sleep onset and offset (range 0.91–1.0). For WASO, inter-rater reliability was more difficult to achieve (range 0.70–0.92); therefore, all WASO codes were reviewed by at least two assistants during monthly coding consensus meetings. All behavioral VSG coders were ultimately reliable at coding WASO (ICC > 0.70) and our consensus practice was an added measure to keep these more difficult codes consistent. Minor and major wakings were post-hoc calculations from WASO minutes.

**Table 2:** Operational definitions of sleep variables.

**Variable**	**Operational definition**
Sleep onset time	The first minute (of at least three consecutive minutes) scored as sleep
Nightime sleep duration	Total minutes scored as sleep from sleep onset time to morning rise time minus any night waking minutes (WASO)
Wake after sleep onset (WASO)	Total minutes scored as awake between sleep onset and offset
Morning rise time (Sleep Offset)	The first five consecutive minutes of awake time, following a period of sleep, when the child is awake for the day
Minor night waking	Night wakings ranging from 1 to 14 min
Major night waking	Night wakings that are 15 min or longer

#### Auto-VSG coding

**Figure 1:**
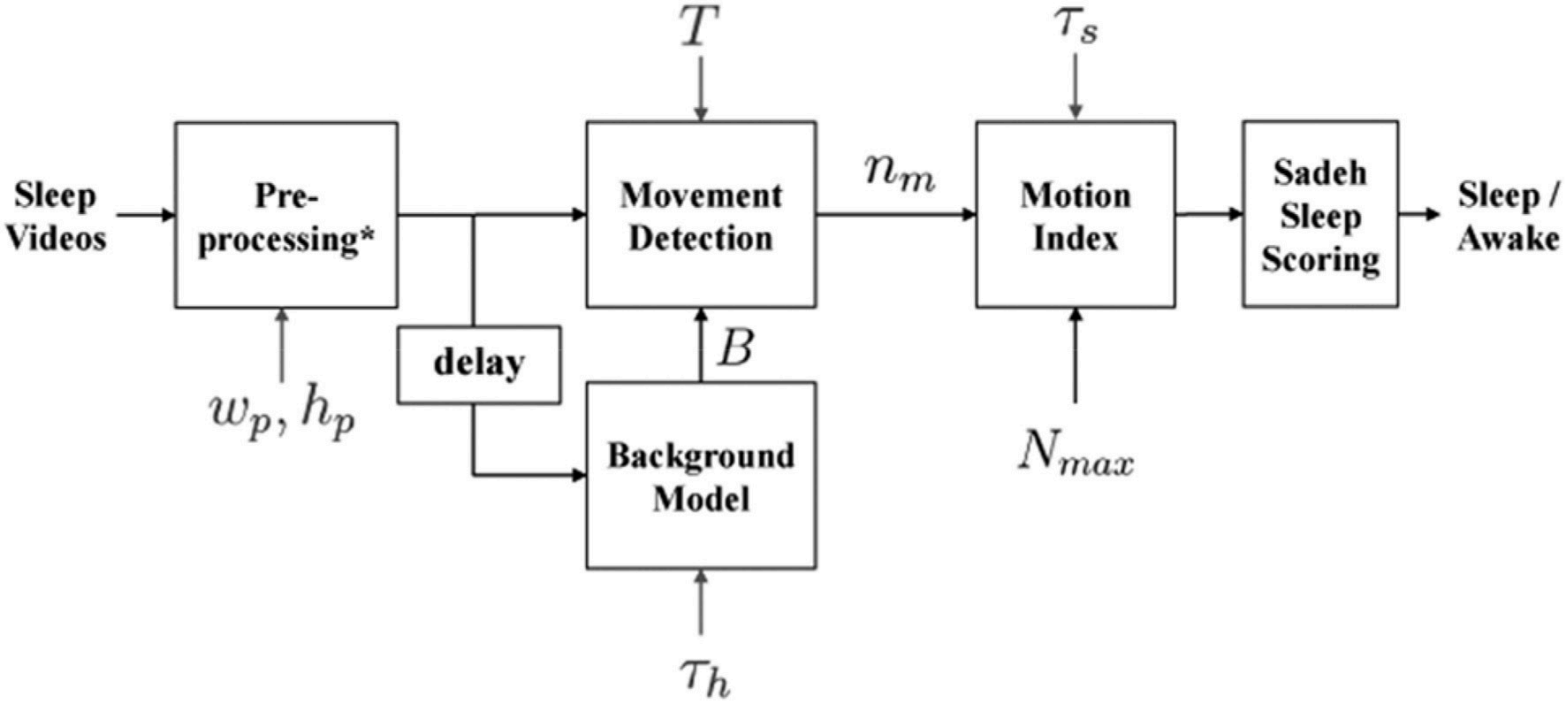
Block diagram of automated videosomnography (auto-VSG) processing system. ^*^Preprocessing block includes Image Resizing, Red Green Blue (RGB) to Gray Scale Conversion and Histogram Equalization. *w*_*p*_ and *h*_*p*_ are width and height of the resized image used in the preprocessing block, τ_*s*_ is duration of each time segment (epoch) [60 s], *h* is the number of frames used to obtain the Background model *B*, *T* is a threshold for each pixel to determine whether there is a movement or not and *n*_*m*_ is number of moved pixels in the current frame.

**Figure 2:**
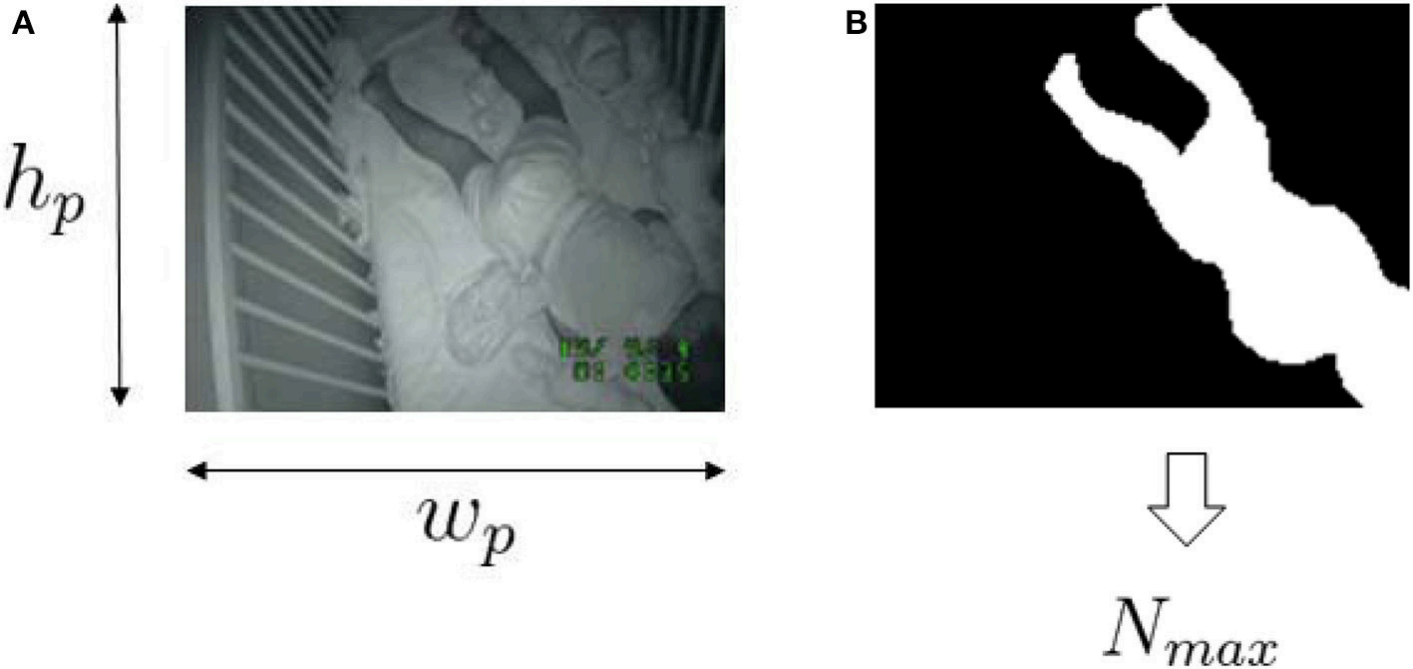
Sample videosomnography frame **(A)** and the corresponding selected infant/toddler area **(B)**. ***w***_*p*_ and ***h***_*p*_ are the width and height of the resized image. *N*_*max*_ is the maximum number of pixels allow to “*move*” within the frame.

To provide automated estimates of sleep onset time, morning rise time (sleep offset), wakings, and nighttime sleep duration, a custom processing systems was employed (detailed in Figures 1, 2). Within this system, infant/toddler movements before, during, and after sleep were assessed using a background subtraction method and a scaled minute-to-minute summary score. These movement scores were then classified as sleep or wake using existing algorithms (21). The decision to index sleep using infant/toddler movement builds on a strong history of using accelerometers or small wrist/ankle worn sensors to estimate sleep using movement (22, 23). Table 2 provides a summary of each auto-VSG system parameter when compared to behavioral VSG and actigraphy.

#### Auto-vidoesomnography system equations

Within our auto-VSG system, we first converted the Red Green Blue (RGB) image to a grayscale image. We then resized the image (*width, w*_*p*_ = 160 *pixels, height, h*_*p*_ = 120 *pixels*) to fit our preprocessing block requirements. The final step in the preprocessing block required histogram equalization, wherein we enhanced the image contrast (i.e., to maximize the discrimination between the infant/toddler and the background). Next, the background model was obtained from the history of *h* previous frames as

(1) $$\begin{array}{r} B_{i}\left[x,y\right]=\frac{1}{h\left[i\right]}\overset{i=1}{\underset{k=i-h\left[i\right]}{\sum }}I_{k}\left[x,y\right] \end{array}$$

where *I*_*i*_[*x, y*] is a pixel in frame *i*, *B*_*i*_[*x, y*] is a pixel in background model at frame *i* and *h*[*i*] is the number of previous frames (history) used for making the background model. The difference between the background model *B*_*i*_[*x, y*] and *I*_*i*_[*x, y*] indicated whether each pixel in the frame was classified as *moved* or *not moved*. This was achieved by comparing the grayscale value across each pixel. A pixel was classified as *moved* if (Equation 2) holds.

(2) $$\begin{array}{r} |I\left[x,y\right]-B\left[x,y\right]|>T \end{array}$$

where *T* is a threshold for determining movement for one pixel. We quantified the amount of movement as the number of pixels classified as *moved*. We obtained the motion index for time segment *j* as:

(3) $$\begin{array}{r} m_{j}=\min\left(\frac{1}{K}\overset{K-1}{\underset{k=0}{\sum }}n_{m}\left[k\right],N_{\;max}\right) \end{array}$$

where *k* is frame index within one epoch, *n*_*m*_[*k*] is number of moved pixels in frame *k*, *N*_max_ is the size of the infant/toddler in the video frame [pixels] and *K* is number of frames for one time segment, ⌊τ_*s*_ · *f*_*s*_⌋ where τ_*s*_ is duration of each time segment [sec]. The motion index is capped at *N*_*max*_ because the largest number of moved pixels that belongs to the infant/toddler's movement would not exceed the size of the infant/toddler. N_*max*_ is obtained from a single frame at midnight by manually annotating the infant/toddler's size. We obtained one N_*max*_ per ID assuming that the change in the number of pixels for the infant/toddler over the entire night is negligible.

We labeled each minute of recording as sleep or wake by applying the Sadeh sleep/wake algorithm designed for actigraphy (21). To apply this algorithm, we scaled the motion index from 0 to 400 (the same range used for the actigraphy data in the present study).

#### Actigraphy

Movements during sleep were recorded in 1 min epochs using a micromini-motionlogger®. Each infant/toddler wore the actigraph on his/her ankle (imbedded in a neoprene band). The actigraph data were interpreted as sleep or wake using the Sadeh algorithm provided in Action-W version 2.7.3. Following published actigraphy guidelines (23), parent-report sleep diaries were used to further interpret our data [including validity; (24)]. Parent-report diaries helped to clarify the usability of data for each recording night. The sleep diary used in the current study is a modified version of previously validated sleep diaries (25, 26), with similar versions used in several published papers [e.g., (6)]. Overall, using a parent-report diary in conjunction with actigraphy data (as described above) is the current field standard (24, 27).

For each actigraph recording night, estimates for sleep onset, sleep offset, nighttime sleep duration, WASO, minor wakings, and major wakings were calculated (Table 2).

### Procedure

This study was approved by the Institutional Review Board of Purdue University. All subjects gave written informed consent in accordance with the Declaration of Helsinki. Families completed the demographic form at time of enrollment. The first home visit included setting up the VSG recording equipment and providing parents with an actigraph and a parent-report sleep diary. Parents were instructed to turn the camera on when they started their infant/toddler's bedtime routine and to turn it off in the morning after the infant/toddler was removed from bed.

### Plan of analysis

To assess the proposed auto-VSG system, we utilized Cohen's kappa, paired sample *t*-tests, Bland-Altman plots, and correlations for estimates of sleep onset, WASO, morning rise time (sleep offset) and nighttime sleep duration across (1) behavioral VSG, (2) auto-VSG, and (3) actigraphy.

First, all synchronized minute-by-minute estimates of sleep vs. wake were compared using Cohen's kappa. Cohen's kappa was employed to index measurement agreement above that expected by chance, while considering the marginal distribution of sleep and wake codes. Paired *t*-tests were used to assess if statistically significant differences existed on average, between the two methods (i.e., behavioral VSG and auto-VSG). To provide an illustrative index of agreement, Bland-Altman plots were generated (28). Additionally, to ground this work within existing studies, correlations were calculated as an index of association. We also interpreted whether differences were clinically meaningful. For example, a difference of 10 min in a 24-h period may be statistically significant but not practically meaningful to clinicians. For minor and major wakings only, descriptive statistics and Bland-Altman plots were employed because most of these count variables were zero-inflated and not normally distributed.

Given the high number of agreement and association statistics assessed in this study, analyses are summarized (**Table 5**) with evaluative (+) supports agreement, (±) modest or mixed support, and (–) does not support agreement. On the basis of these results, we assigned qualitative ratings of strong, moderate, or poor agreement.

For all analyses, auto-VSG was compared to behavioral VSG and actigraphy, separately because auto-VSG estimates were calculated using different N_*max*_ settings (see Table 3). Additionally, recognizing that the focus of the current study is auto-VSG, the inclusion of comparisons between actigraphy to behavioral VSG are not discussed as these results are beyond the scope of the current study.

**Table 3:** Automatic videosomongraphy (auto-VSG) system parameters.

**Parameter**	**Description**	**Behavioral VSG settings**	**Actigraphy settings**
*w_*p*_*	Width of resized image	160 pixels	160 pixels
*h_*p*_*	Height of resized image	120 pixels	120 pixels
T	Color intensity differencing threshold	30 levels (11.76% of the color intensity)	30 levels (11.76% of the color intensity)
τ_*s*_	Length of epoch	60 s	60 s
τ_*h*_	Time used to build background model	5 s	5 s
*N_max_*	The maximum number of pixels that could contribute to the activity count	100%	15%

## Results

### Behavioral VSG and Auto-VSG

Kappa estimates of sleep vs. wake ranged from 0.09 (poor) to 0.99 (strong) with an average agreement of 0.46 (moderate). For three participants, behavioral and auto-VSG demonstrated very strong agreement (kappa > 0.99) but most (*n* = 25) had strong to moderate agreement, and for two participants, poor agreement (kappa < 0.10). When using the behavioral VSG codes as the *true* codes, auto-VSG demonstrated sensitivity of 99% to correctly classify sleep, with specificity of 48%. In this case, specificity indexed when a minute was coded as wake using behavioral VSG and auto-VSG, respectively. Overall, auto-VSG identified sleep with a higher degree of accuracy than wake (approximately half of the wake minutes were coded correctly with auto-VSG).

#### Sleep onset time

**Figure 3:**
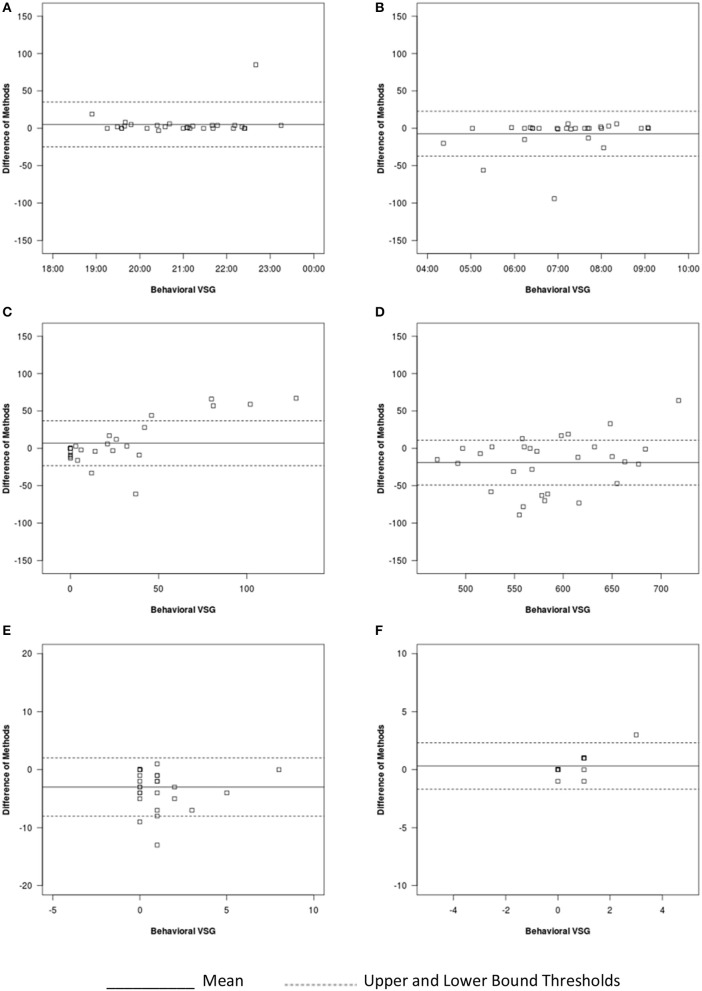
Bland-Altman plots for auto-VSG and behavioral VSG coding for **(A)** sleep onset, **(B)** sleep offset, **(C)** WASO, **(D)** sleep duration, **(E)** number of minor wakings, and **(F)** number of major wakings.

For sleep onset time, there was no significant difference between behavioral VSG and auto-VSG estimates (Table 4). The Bland-Altman plot for this data (Figure 3A) illustrates strong agreement, with only 3% of the sample falling outside the target threshold. Overall, for sleep onset time, behavioral VSG and auto-VSG demonstrated strong agreement (Table 5).

**Table 4:** Paired-sample *t*-test for automated videosomnography (auto-VSG), behavioral VSG, and actigraphy.

	**Sleep onset time**	**Sleep offset time**	**WASO**	**Sleep duration**	**Number of minor wakings**	**Number of major wakings**
	**M (SD) [HH:MM]**	**M (SD) [HH:MM]**	**M (SD) [minutes]**	**M (SD) [minutes]**	**M (SD)**	**M (SD)**
**Auto-VSG and Behavioral VSG**
Behavioral VSG	20:59 (1:10)	7:09 (1:08)	24.77 (33.37)	585.83 (60.71)	1.00 (1.72)	0.47 (0.68)
Auto-VSG	20:53 (1:08)	7:15 (1:06)	17.43 (22.40)	605.47 (61.53)	3.87 (3.82)	0.13 (0.43)
Paired *t*-test	*t*_(29)_ = 1.97	*t*_(29)_ = −1.79	*t*_(29)_ = 1.42	*t*_(29)_ = −3.03**		
**Auto-VSG and Actigraphy**
Actigraphy	21:00 (1:02)	6:49 (1:09)	129.67 (54.36)	459.23 (60.57)	21.87 (8.41)	2.10 (1.79)
Auto-VSG	20:59 (1:09)	7:01 (1:07)	145.30 (85.60)	457.50 (66.49)	22.77 (10.44)	2.40 (2.49)
Paired *t*-test	*t*_(29)_ = 0.42	*t*_(29)_ = −2.68*	*t*_(29)_ = −1.47	*t*_(29)_ = 0.14		

All estimates were generated based on 18,943 min of synchronized data (across all sleep assessment methods). Differences across the two Auto-VSG methods reflect differences in system parameters (summarized in Table 3) *p < 0.05, **p < 0.01.

**Table 5:** Summary of measurement agreement wherein each test statistic was evaluated with (+) supports agreement, (+/−) mixed or inconsistent support, (−) poor support.

	***R***	***t*-test**	**Bland-Altman Plot^a^**	**Clinically meaningful difference^b^**	**Overall agreement**
**Behavioral VSG and auto-VSG**
Sleep Onset Time	0.97^**^(+)	+	97% (+)	6 min (+)	Strong
Sleep Offset Time	0.95^**^(+)	+	93% (+)	6 min (+)	Strong
WASO	0.55^**^(±)	+	77% (±)	7 min (+)	Moderate
Sleep Duration	0.83^**^(+)	–	62% (−)	20 min (−)	Poor
**Actigraphy and auto-VSG**
Sleep Onset Time	0.97^**^(+)	+	93% (+)	1 min (+)	Strong
Sleep Offset Time	0.93^**^(+)	–	80% (±)	12 min (−)	Poor
WASO	0.75^**^(+)	+	53% (−)	16 min (−)	Poor
Sleep Duration	0.41^*^(−)	+	50% (−)	2 min (+)	Poor

r, correlation, t-test results summarized in Table 3,

a % of sample correctly estimated within the upper and lower bound thresholds, see Bland-Altman plots,

b average difference across methods,

* p < 0.05,

** *p < 0.01*.

#### Sleep offset time

For sleep offset time, behavioral VSG and auto-VSG estimates were comparable for both methods. As illustrated in the Bland-Altman plot (Figure 3B) and Table 4, most infants/toddlers fell within the established target threshold (93%). Overall, for sleep offset time behavioral VSG and auto-VSG had strong agreement.

#### Night waking

The *t-*tests supported comparable estimates across behavioral VSG and auto-VSG for WASO. However, the Bland-Altman plot depicts a potential relationship between measurement agreement and WASO duration (Figure 3C). There was less agreement across the measures for infants/toddlers who were awake more after sleep onset. Overall behavioral VSG and auto-VSG had moderate agreement WASO (Table 5). When considering minor and major wakings, distributional properties of these data did not allow for the same type of comparisons. However, visual inspection of these data (Figures 3E,F) illustrate that for most children agreement across behavioral VSG and auto-VSG was moderate.

#### Nighttime sleep duration

Nighttime sleep duration estimates were significantly different across the behavioral VSG and auto-VSG processing methods (Table 4). The Bland-Altman plot (Figure 3D) highlight the large levels of variability in the agreement across these measures. Additionally, only 62% of infants/toddlers fell within the target threshold. For nighttime sleep duration, behavioral VSG and auto-VSG demonstrated poor agreement.

### Actigraphy and Auto-VSG

Overall, kappa estimates for sleep vs. wake ranged from 0.03 (poor) to 0.82 (strong) with an average of 0.41 (moderate). For three participants, actigraph and auto-VSG demonstrated strong agreement (kappa > 0.70) but most (*n* = 24) had moderate agreement, and for three participants poor agreement (kappa < 0.10). When using the actigraphy minute-by-minute codes as the *true* score, the auto-VSG codes demonstrated a sensitivity of 84% to correctly classify sleep and a specificity of 58%. Overall, auto-VSG identified sleep with a higher degree of accuracy than wake (approximately half of the wake minutes were coded correctly with auto-VSG).

#### Sleep onset time

**Figure 4:**
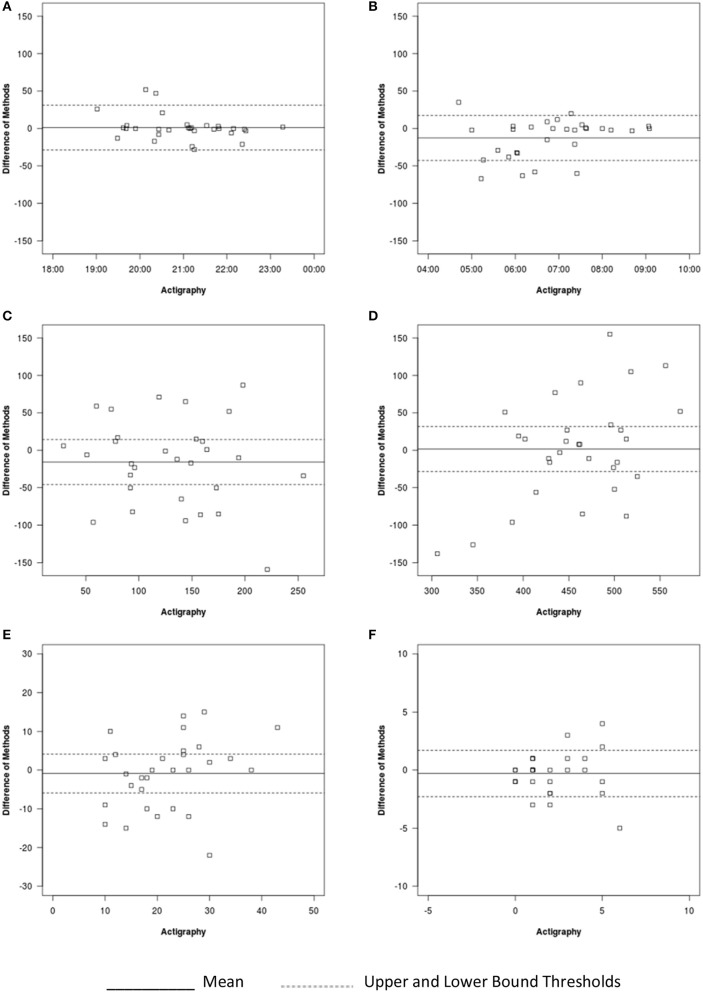
Bland-Altman plots for auto-VSG and actigraphy for **(A)** sleep onset, **(B)** sleep offset, **(C)** WASO, **(D)** sleep duration, **(E)** number of minor wakings, and **(F)** number of major wakings.

For sleep onset time, there was no significant difference between actigraphy and auto-VSG estimates (Table 4). Only data from two infants/toddlers (7% of sample), resulted in measurement agreement outside the target threshold (Figure 4A). Actigraphy and auto-VSG demonstrated strong agreement for sleep onset time (Table 5).

#### Sleep offset time

On average, sleep offset time was significantly different across the two methods (Table 4). This difference was on average 12 min, with earlier offset times provided by actigraphy. The Bland-Altman plot revealed that data from six infants/toddlers (20%) had measurement agreement outside the target threshold (Figure 4B). Overall, actigraphy and auto-VSG demonstrated poor agreement for sleep offset time (Table 5).

#### Night waking

WASO was not significantly different across the two methods (Table 4); however, within the Bland-Altman plot, only 53% of sample had measurement agreement inside the target threshold (Figure 4C). For WASO, actigraphy and auto-VSG demonstrated poor agreement (Table 5). Visual inspection of the minor (Figure 4E) and major (Figure 4F) wakings data illustrate that for most children agreement across actigraphy and auto-VSG was poor.

#### Nighttime sleep duration

For nighttime sleep duration, average estimates were not significantly different across the two methods (Table 4); however, as illustrated in the Bland-Altman plot (Figure 4D), half of the infants/toddlers did not fall within the specified threshold. Overall, actigraphy and auto-VSG demonstrated poor agreement for nighttime sleep duration (Table 5).

## Discussion

The automation of VSG coding has the potential to improve research paradigms and expand its clinical applications. In the current study, we demonstrated initial support for auto-VSG to index sleep timing in a pediatric sample, with the strongest agreement for sleep onset and offset when compared to behavioral VSG. However, the overall agreement patterns is more complex as discussed in the following sections.

Sleep problems are the most common concern expressed by parents at well-child exams (29) and for many families, these sleep concerns reflect clinically meaningful sleep disturbances. Mass market auto-VSG devices like Nanit®, AngelCare®, BabbyCam®, and Knit Health are well poised to build on these concerns; however, rigorous studies are needed before clinical applications or interpretation are advisable. The processing system tested within the current study builds on an existing signal/video-processing approach (background subtraction) and does not directly represent the systems used in the above noted mass market systems. However, the present study provides preliminary information on the use of auto-VSG, and with replication (and likely system improvements), it may be refined to further index infant/toddler sleep.

With the rise of telemedicine approaches, clinicians desire easy-to-use tools that may bridge the gap from clinic to in-home assessments. Auto-VSG has the potential to provide an index of infant/toddler sleep timing within the comfort of an infant/toddler's home environment—without the use of electrodes, or wrist/ankle monitors. Additionally, when implementing clinical recommendations, video platforms may allow parents to actively monitor their infant/toddler's sleep. However, the results of this study are preliminary and only provide initial support for its use in estimating sleep timing (sleep onset and offset) and not for wakings or nighttime sleep duration.

The lack of agreement between auto-VSG and actigraphy within the present study could reflect several factors. For example, actigraphy has demonstrated poor sleep/wake specificity in certain populations, including young adults and school-age children when compared to PSG (30, 31). Additionally, actigraphy estimates may be less accurate in children with particularly elevated sleep problems (32). In the current study, close inspection of the infants/toddlers with the highest levels of disagreement between acitgraphy and auto-VSG revealed that for most of these children, actigraphy was overestimating WASO. Although videos clearly indicated the infants/toddlers were moving, these movements did not culminate into wakings. Similar errors have been observed in previous research (25, 33, 34). Additionally, our multifaceted analytic approach, while adding methodical rigor, may also be contributing to our incongruent results (when compared to other studies). For example, Mantua et al. (35) assessed agreement between PSG and several actigraphy devices and reported high correlations for many of their comparisons; however, as the current study demonstrates, high correlations do not equate high agreement. Finally, the placement of our actigraph device (ankle band) may have influenced our results because at least one previous study documented the highest agreement between actigraphy and PSG with a wrist placement (36). However, clinical recommendations for actigraphy placement in young children include the ankle (23). Additionally, actigraph ankle placement is generally tolerated better in young children. Within the present study, we opted for ankle placement to maximize child compliance.

The interpretation of auto-VSG in this study builds heavily on the movement assumptions of sleep. Like with actigraphy, this study used infant/toddler movement as a proxy for sleep, recognizing that sleep with no movement may reflect deep sleep or slow wave sleep. Although this assumption is common is developmental sleep research (22), it is important to note that lack of movement is not the same as sleep. The proposed auto-VSG system suffers from the same limitations as actigraphy with respect to sleep estimates based on movement. This limitation likely contributed to the low agreements for waking, as have been documented in actigraphy [e.g., (25)]. Similarly, this may be why sleep onset and morning rise time agreements were higher as they represent the strongest shifts from no movement to movement or vice versa. If PSG were incorporated, sleep onset and morning rise times would likely have acceptable agreement for this reason. Additionally, it is probable that estimates for slow wave sleep (given its low movement profile) would be acceptable but estimates of Non-REM Stage 1, 2, and REM sleep would likely be low.

There are several notable limitations to the clinical applications of the auto-VSG system tested within this study. First, the sleep parameters assessed reflect only a portion of concerns faced by clinicians. When assessing pediatric sleep concerns, more information regarding sleep behaviors (e.g., REM sleep, seizure activity) may be warranted. However, real-time videos can provide clinicians with unique information regarding parenting behaviors and the sleep environment (e.g., light, noise, and other ambient features). Additionally, the tested auto-VSG system poorly characterized sleep for only some of the infant/toddlers in this study. Further research is needed to identify why some infants/toddlers were accurately captured and others were not. Finally, the use or generalization of the data from this study is limited by the relatively small sample size. Although, to our knowledge, this is the largest study of auto-VSG, replication is needed before clinical implementation is recommended.

Future studies of auto-VSG systems can build on this study in several ways. First, the classification of infant/toddler sleep as sleep or wake is an oversimplification and future studies should code for either sleep stages or consider adding active and quiet sleep states. Infant/toddlers transition in and out of sleep pass through several “phases” that are often indeterminate. This likely reflects why waking variables had the lowest agreements. Additionally, future signal processing systems should incorporate all available data including the audio signal. Incorporating the audio signal may allow for more nuanced codes of wake settled or wake distressed and may help disentangle intermediate sleep states. Future studies may also build on this work by applying the presented auto-VSG system to PSG and to larger, more diverse samples. Additionally, improvements in the presented auto-VSG system may result in more accurate sleep and wake estimates.

In sum, with recent technological advancements, auto-VSG is feasible and as demonstrated in the current study may provide relatively comparable estimates to behavioral VSG for sleep timing.

## Author contributions

AS served as the principal investigator and was a substantial contributor to each piece of the project. ED and JC developed the signal processing system and completed most of the statistical analyses with AS. AK and EA assisted with the data collection and preparation. Additionally, all authors assisted in writing and revising sections of the manuscript.

### Conflict of interest statement

The authors declare that the research was conducted in the absence of any commercial or financial relationships that could be construed as a potential conflict of interest.

## References

[1] Anders TF Keener M. Developmental course of nighttime sleep-wake patterns in full-term and premature infants during the first year of life: I. Sleep (1985) 8:173–92. 10.1093/sleep/8.3.173 4048734

[2] Merlino G Serafini A Dolso P Canesin R Valente M Gigli GL. Association of body rolling, leg rolling, and rhythmic feet movements in a young adult: a video-polysomnographic study performed before and after one night of clonazepam. Mov Disord. (2008) 23:602–607. 10.1002/mds.21902 18175344

[3] Sadeh A III. Sleep assessment methods. Monogr Soc Res Child Dev. (2015) 80:33–48. 10.1111/mono.12143 25704734

[4] Batra EK Teti DM Schaefer EW Neumann BA Meek EA Paul IM. Nocturnal video assessment of infant sleep environments. Pediatrics (2016) 138: e20161533. 10.1542/peds.2016-1533 27527797 PMC5005029

[5] Giganti F Fagioli I Ficca G Cioni G Salzarulo P. Preterm infants prefer to be awake at night. Neurosci Lett. (2001) 312:55–7. 10.1016/S0304-3940(01)02192-9 11578844

[6] Schwichtenberg AJ Hensle T Honaker S Miller M Ozonoff S Anders T. Sibling sleep-What can it tell us about parental sleep reports in the context of autism? Clin Pract Pediatr Psychol. (2016) 4:137–52. 10.1037/cpp0000143 27563509 PMC4993283

[7] St. James-Roberts I Roberts M Hovish K Owen C. Descriptive figures for differences in parenting and infant night-time distress in the first three months of age. Prim Health Care Res Dev. (2016) 17:611–21. 10.1017/S1463423616000293 27609027 PMC5356193

[8] Teti DM Crosby B. Maternal depressive symptoms, dysfunctional cognitions, and infant night waking: the role of maternal nighttime behaviour. Child Dev. (2012) 83:939–953. 10.1111/j.1467-8624.2012.01760.x 22506917

[9] Tipene-Leach D Baddock S Williams S Tangiora A Jones R McElnay C Taylor B. The Pēpi-Pod study: overnight video, oximetry and thermal environment while using an in-bed sleep device for sudden unexpected death in infancy prevention. J Paediatr Child Health (2018) 54:638–46. 10.1111/jpc.13845 29357197

[10] Shimohira M Shiiki T Sugimoto J Ohsawa Y Fukumizu M Hasegawa T. Video analysis of gross body movements during sleep. Psychiatry Clin Neurosci. (1998) 52:176–7. 10.1111/j.1440-1819.1998.tb01015.x 9628136

[11] Liao WC Chiu MJ Landis CA. A warm footbath before bedtime and sleep in older Taiwanese with sleep disturbance. Res Nurs Health (2008) 31:514–28. 10.1002/nur.20283 18459154 PMC2574895

[12] Okada S Ohno Y Goyahan, Kato-Nishimura K Mohri I Tanike M. Examination of non-restrictive and non-invasive sleep evaluation technique for children using difference images. Conf Proc IEEE Eng Med Biol Soc. (2008) 2008:3483–7. 10.1109/IEMBS.2008.4649956 19163459

[13] Yang CM Wu CH Hsieh MH Liu MH Lu FH. Coping with sleep disturbances among young adults: a survey of first-year college students in Taiwan. Behav Med. (2003) 29:133–8. 10.1080/08964280309596066 15206832

[14] Okada S Shiozawa N Makikawa M Body movement in children with adhd calculated using video images. In: Proceedings of the IEEE 35th Annual International Conference on Engineering in Medicine and Biology Society. Hong Kong (2012). p. 60–1. 10.1109/BHI.2012.6211505

[15] Nakatani M Okada S Shimizu S Mohri I Ohno Y Taniike M. Body movement analysis during sleep for children with adhd using video image processing. Conf Proc IEEE Eng Med Biol Soc. (2013) 2013:6389–92. 10.1109/EMBC.2013.6611016 24111203

[16] Wang CW Hunter A A simple sequential pose recognition model for sleep apnea. In: Proceedings of the IEEE 8th Annual International Conference on Bioinformatics and Bioengineering. Athens (2008). p. 938–43. 10.1109/BIBE.2008.4696808

[17] Wang CW Hunter A A robust pose matching algorithm for covered body analysis for sleep apnea. In: Proceedings of the IEEE 8th Annual International Conference on Bioinformatics and Bioengineering Athens (2008). p. 1158–64. 10.1109/BIBE.2008.4696847

[18] Glazer A Systems and Methods for Configuring Baby Monitor Cameras to Provide Uniform Data Sets for Analysis and to Provide an Advantageous View Point of Babies. Patent US 20150288877 A1. (2015). (Accessed October 8, 2015).

[19] Heinrich A Aubert X de Haan G Body movement analysis during sleep based on video motion estimation. In Proceedings of the IEEE 15th International Conference on e-Health Networking, Applications and Services. Lisbon (2013) p. 539–43. 10.1109/HealthCom.2013.6720735

[20] Albanese A,. Bedtime Story: Deep Learning Baby Monitor Keeps an Eye on Your Crib. (2016). Available online at: https://blogs.nvidia.com/blog/2016/10/30/babbycam-baby-monitor-deep-learning (Accessed November 28, 2016).

[21] Sadeh A. Assessment of intervention for infant nigh waking – parental reports and activity-based home monitoring. J Consult Clin Psychol. (1994) 62:63–9. 10.1037/0022-006X.62.1.63 8034831

[22] Acebo C LeBourgeois MK. Actigraphy. Respir Care Clin N Am. (2006) 12:23–30. 10.1016/j.rcc.2005.11.010 16530645

[23] Ancoli–Israel S Martin JL Blackwell T Liu L Taylor DJ. The SBSM guide to actigraphy monitoring: clinical and research applications. Behav. Sleep Med. (2015) 13:S4–S38. 10.1080/15402002.2015.1046356 26273913

[24] Meltzer LJ Montgomery-Downs HE Insana S Walsh CM. Use of actigraphy in pediatric sleep research. Sleep Med Rev. (2012) 16:463–75. 10.1016/j.smrv.2011.10.002 22424706 PMC3445439

[25] So K Adamson TM Horne RS. The use of actigraphy for assessment of the development of sleep/wake patterns in infants during the first 12 months of life. Sleep Res. (2007) 16:181–7. 10.1111/j.1365-2869.2007.00582.x 17542948

[26] Wolfson AR Carskadon MA Acebo C Seifer R Fallone G Labyak SE Martin JL. Evidence for the validity of a sleep habits survey for adolescents. Sleep (2013) 26:213–6. 10.1093/sleep/26.2.213 12683482

[27] Horne RSC Biggs SN Actigraphy and sleep/wake diaries. In: Wolfson A, Montgomery-Downs H, editors. The Oxford Handbook of Infant, Child, and Adolescent Sleep and Behavior. New York, NY: Oxford University Press (2013). p. 189–203.

[28] Bland JM Altman DG Statistical methods for assessing agreement between two methods of clinical measurement. Lancet (1986) 327:307–10. 10.1016/S0140-6736(86)90837-8 2868172

[29] Meltzer LJ McLaughlin Crabtree V Pediatric Sleep Problems: A Clinician's Guide to Behavioural Interventions. Washington, DC: American Psychological Association (2015).

[30] Meltzer LJ Wong P Biggs SN Traylor J Kim JY Bhattacharjee R. Validation of actigraphy in middle childhood. Sleep (2016) 39:1219–24. 10.5665/sleep.5836 27091520 PMC4863209

[31] Rupp TL Balkin TJ. Comparison of motionlogger watch and actiwatch actigraphs to polysomnography for sleep/wake estimation in healthy young adults. Behav Res Methods (2011) 43:1152–60. 10.3758/s13428-011-0098-4 21512871

[32] Sadeh A. The role and validity of actigraphy in sleep medicine: an update. Sleep Med Rev. (2011) 15:259–67. 10.1016/j.smrv.2010.10.001 21237680

[33] Gertner S Greenbaum CW Sadeh A Dolfin Z Sirota L Ben-Nun Y. Sleep-wake patterns in preterm infants and 6 month's home environment: implications for early cognitive development. Early Hum Dev. (2002) 68:93–102. 10.1016/S0378-3782(02)00018-X 12113995

[34] Gnidovec B Neubauer D Zidar J. Actigraphic assessment of sleep-wake rhythm during the first 6 months of life. Clin Neurophysiol. (2002) 113:1815–21. 10.1016/S1388-2457(02)00287-0 12417236

[35] Mantua J Gravel N Spencer RMC. Reliability of sleep measures from four personal health monitoring devices compared to research-based actigraphy and polysomnography. Sensors (2016) 16:646. 10.3390/s16050646 27164110 PMC4883337

[36] Ray MA Youngstedt SD Zhang H Robb SW Harmon BE Jean-Louis G. Examination of wrist and hip actigraphy using a novel sleep estimation procedure. Sleep Sci. (2014) 7:74–81. 10.1016/j.slsci.2014.09.007 25580202 PMC4286157

